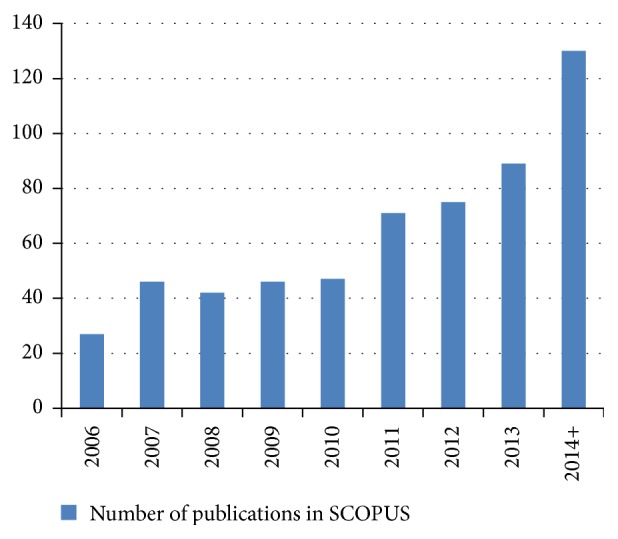# Mechanisms, Efficacy, and Safety of *Bacopa monnieri* (Brahmi) for Cognitive and Brain Enhancement

**DOI:** 10.1155/2015/717605

**Published:** 2015-08-30

**Authors:** Con Stough, Hemant Singh, Andrea Zangara

**Affiliations:** ^1^Centre for Human Psychopharmacology, Swinburne University, P.O. Box 218, Hawthorn, VIC 3122, Australia; ^2^Central Drug Research Institute, Lucknow, Uttar Pradesh 226031, India; ^3^Lumen Research Foundation, No.2, 1st Cross Street, 2nd Avenue, Chennai, Tamil Nadu 600083, India; ^4^NaturalPowerMeds Consulting SL, 08028 Barcelona, Spain

The plant* Bacopa monnieri* (water hyssop, Brahmi, thyme-leaved* Gratiola*, herb of grace, and Indian pennywort) is a perennial, creeping herb native to the wet lands of India, particularly northeast and southern regions.* Bacopa* is an important plant of Ayurveda, where it is named as Brahmi, after Lord Brahma, the mythological creator of the world and originator of the science of Ayurveda.* Bacopa* is frequently mentioned in the religious, social, and medical treatises of India since the time of* Vedic* civilization. Its antiquity can be traced to the time of* Athar Ved* (the science of well-being) written in 800 BC where* Bacopa* finds a mention in the very first verse of the third chapter of* Athar Samhita* (compilation on the factors promoting well-being).

More recently researchers have turned their attention to better understanding the mechanisms and efficacy of various extracts of* Bacopa monnieri* on human conditions. Although extracts of* Bacopa* have been studied and used to treat various disorders for centuries (pain, epilepsy, and inflammation, amongst many) perhaps the chief therapeutic claim concerning its benefits has been in improving memory. The Indian government has invested significant resources and conducted hundreds of studies examining the mechanisms of action on the brain and at a cellular level. Interestingly this research has uncovered a myriad of possible mechanisms relating to anti-inflammatory, antioxidant, metal chelation, amyloid, and cholinergic effects amongst many others. Although it is not unusual for plant based medicines to have multiple effects on cellular processes,* Bacopa monnieri* is perhaps one of the most scientifically studied in terms of mechanisms of action. Interestingly these mechanisms seem to comprehensively map on to the biological mechanisms that many researchers have argued underpin cognitive and memory processes. In 1996 a special extract of* Bacopa monnieri* was launched by the Indian Government's Central Drug Research Institute, Lucknow, termed CDRI 08. It was thought at the time that this particular standardised extract had been subjected to the most research and was the most promising extract for medical conditions. In 2010 the three editors for this special issue attended CDRI's 60th research anniversary where a special one-day symposium on research on CDRI 08 was held. It is this extract of* Bacopa monnieri* that is the focus of most of the papers in this special issue which reports studies relating to the safety, mechanisms, and efficacy of specific extracts of* Bacopa monnieri*.

Over the last ten years there have been growing scientific studies on this interesting terrestrial herb. As can be seen in Figure 1 the number of publications concerning* Bacopa monnieri* is steadily growing reflecting increasing scientific interest in this plant for human conditions. Most of these studies reflect scientific endeavours relating to cellular mechanisms. As such these studies are an excellent base to launch larger clinical trials in humans. Although much is known about the mechanisms of* Bacopa* extracts on the brain there are still significant gaps in our knowledge. For instance, long-term chronic trials in older people are now required to understand whether* Bacopa* extracts such as CDRI 08 can prevent age-related cognitive decline or even more insidious diseases such as Alzheimer's dementia. We note that a number of studies are also currently examining the effect of* Bacopa* extracts on improving cognitive and behavioural function in younger people. Clearly the next decade will focus on larger clinical trials in humans and expand upon the excellent animal and preclinical work mainly conducted in India.

*Con Stough* *Con Stough* *Hemant Singh* *Hemant Singh* *Andrea Zangara* *Andrea Zangara*

## Figures and Tables

**Figure 1:**